# A case of effective intravenous methylprednisolone pulse therapy against severe COVID‐19 infection after arteriovenous graft surgery

**DOI:** 10.1002/rcr2.889

**Published:** 2021-12-17

**Authors:** Sokichi Kamata, Souhei Hamanaka, Jin Tsukamoto, Teppei Tsuchimoto, Kiyohiro Fujiwara, Mikio Okamura

**Affiliations:** ^1^ Department of Cardiovascular Surgery Naniwa Ikuno Hospital Osaka Japan; ^2^ Department of Cardiovascular Surgery Moriguchi Ikuno Memorial Hospital Osaka Japan; ^3^ Department of Nephrology Naniwa Ikuno Hospital Osaka Japan; ^4^ Department of Anesthesiology Naniwa Ikuno Hospital Osaka Japan; ^5^ Department of Respiratory Medicine Naniwa Ikuno Hospital Osaka Japan

**Keywords:** COVID‐19 infection, end‐stage renal disease, steroid pulse therapy

## Abstract

Perioperative COVID‐19 infections in patients suffering from end‐stage renal disease (ESRD) are more likely to become severe, with a high mortality rate, than those in other patients. For such patients, corticosteroid therapy is one of the limited number of treatment options. We experienced a case of ESRD in which COVID‐19 infection immediately followed arteriovenous graft surgery. Although the respiratory condition deteriorated following dexamethasone administration, requiring invasive mechanical ventilation, intravenous methylprednisolone pulse therapy (pulse therapy) improved it dramatically, suggesting that pulse therapy may be effective against severe COVID‐19 infection in patients suffering from ESRD.

## Graphical abstract

COVID‐19 infections in patients suffering from end‐stage renal disease (ESRD) are more likely to become severe than in other patients, with a high mortality rate. Among the therapeutic agents known to be effective, only corticosteroids can be used in patients suffering from ESRD, significantly limiting treatment options. We report a case of ESRD in which COVID‐19 infection immediately followed arteriovenous graft surgery. Although the respiratory condition deteriorated following dexamethasone administration, requiring invasive mechanical ventilation, pulse therapy was successful.

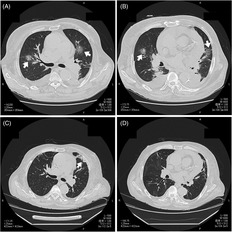

## INTRODUCTION

COVID‐19 infections in patients suffering from end‐stage renal disease (ESRD) are more likely to become severe than in other patients, with a high mortality rate.^1^ Among the therapeutic agents known to be effective, only corticosteroids can be used in patients suffering from ESRD, significantly limiting treatment options. While low‐dose corticosteroid therapy is recommended,^2^ the efficacy and safety of high‐dose corticosteroid therapy with intravenous methylprednisolone pulse therapy (pulse therapy) have not been confirmed. We experienced a case of ESRD in which COVID‐19 infection immediately followed arteriovenous graft (AVG) surgery. Although the respiratory condition deteriorated following dexamethasone administration, requiring invasive mechanical ventilation, pulse therapy was successful. We herein report the findings of this case along with bibliographic considerations.

## CASE REPORT

**FIGURE 1:**
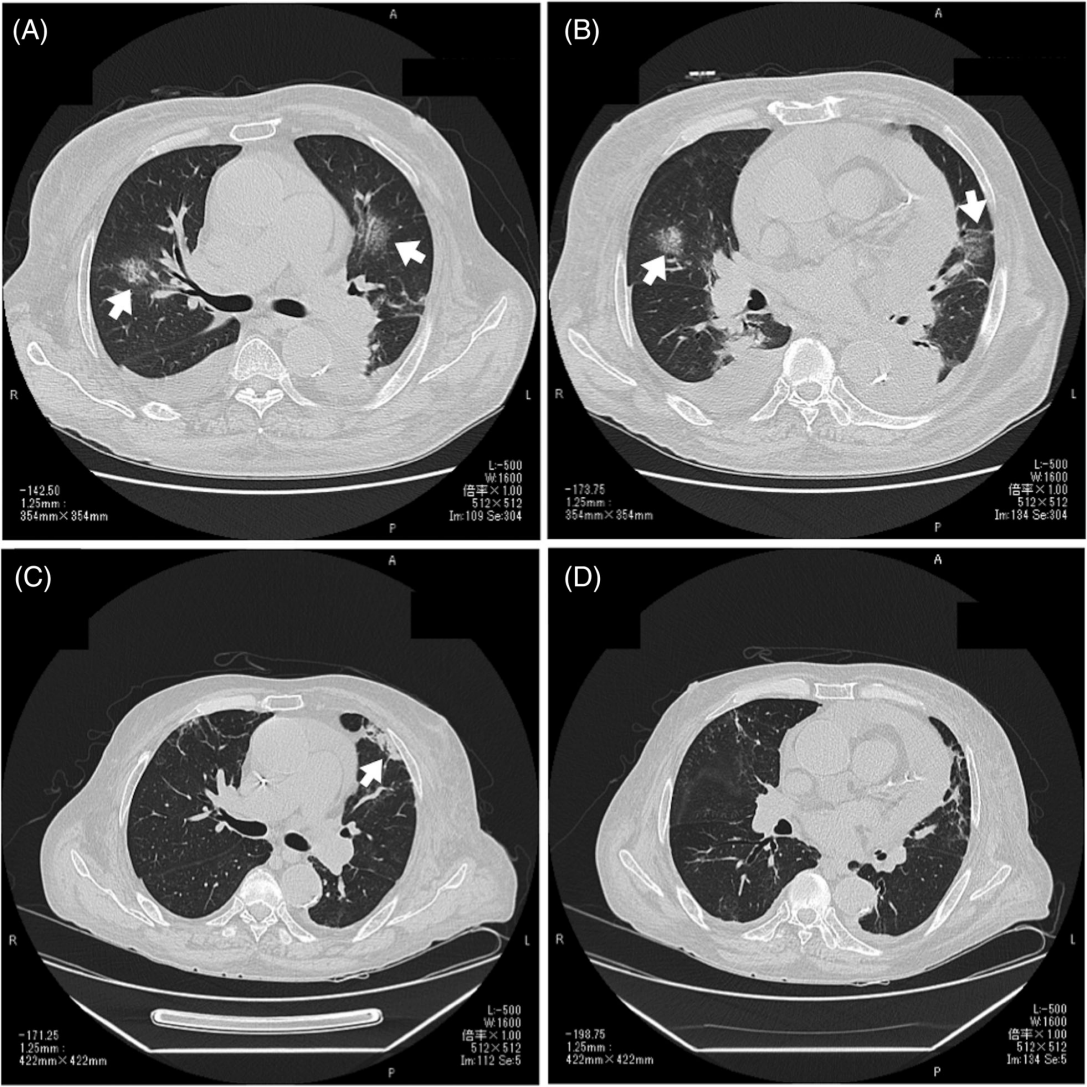
Postoperative day 3 chest computed tomography (CT) (A, B) showed ground‐glass‐like patchy shadows (white arrows), pericardial fluid and bilateral pleural effusion in both lungs. On postoperative day 28 chest CT (C, D), pleural effusion was decreased compared to the onset of COVID‐19 infection, with only mild post‐pneumonia fibrosis (white arrow) observed in the upper left lobe

**FIGURE 2:**
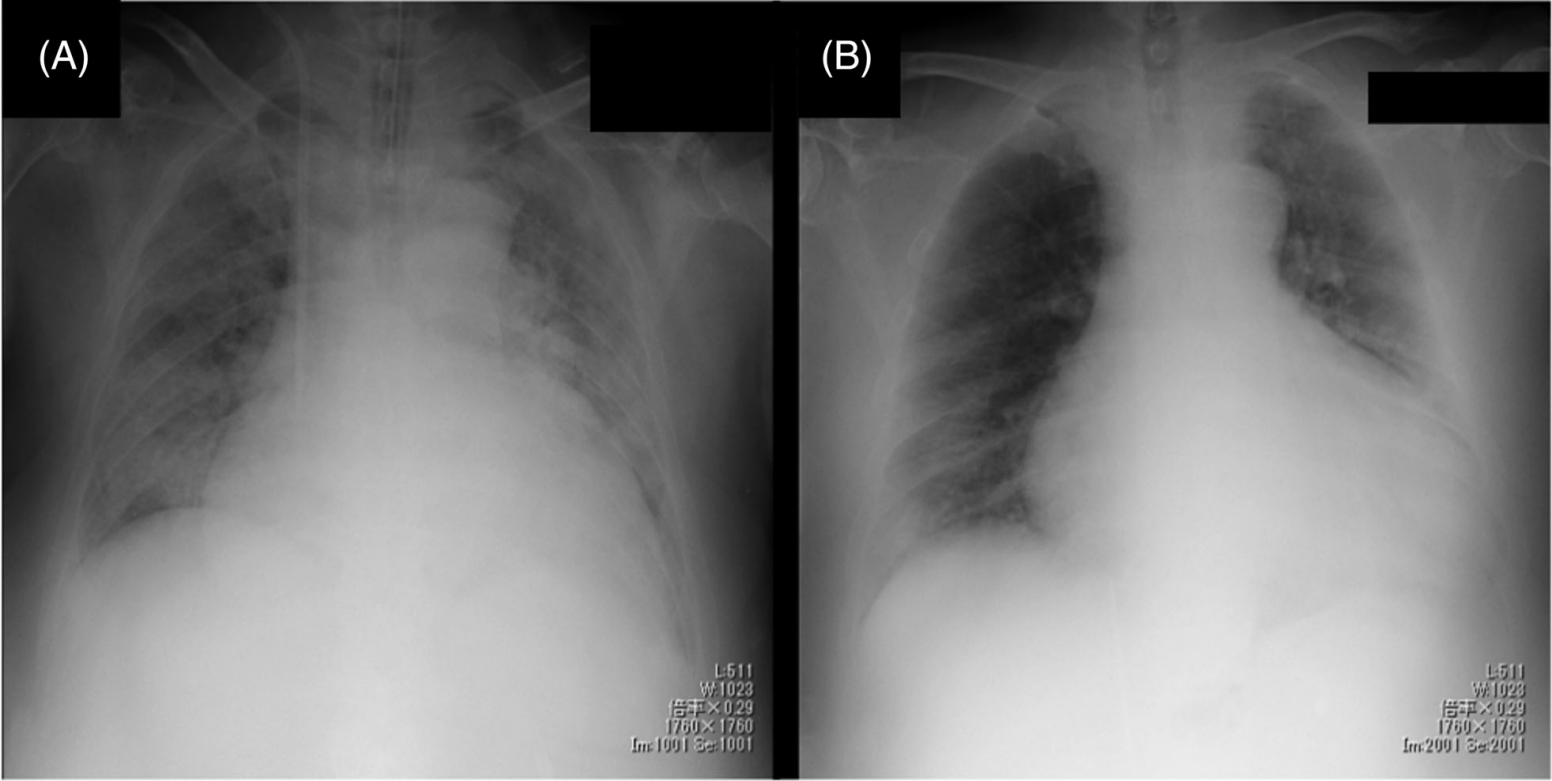
Although granular shadows (A) were observed throughout the entire lung field due to exacerbation of the COVID‐19 infection, improved permeability (B) was observed using pulse therapy

A 66‐year‐old man developed diverticulitis 7 years ago, at which time mild renal dysfunction was noted. His renal function gradually deteriorated, and he was treated twice in the hospital, with anasarca observed 6 months ago. Shortness of breath upon exertion appeared 2 weeks ago. Laboratory tests revealed an elevated serum creatinine level of 7.08 mg/dl and a decreased haemoglobin level of 6.5 mg/dl. Therefore, the creation of vascular access and initiation of dialysis were considered necessary. As our facility was under a state of emergency, reverse transcription polymerase chain reaction (PCR) with a nasopharyngeal swab was performed at admission, showing negative results. Systemic oedema and anaemia were severe, so he received loop diuretics for oedema treatment and blood transfusion of 6 units of packed red blood cells prior to surgery. One week after admission, AVG surgery was performed under laryngeal mask anaesthesia combined with intravenous sedation. On day 1 following surgery, fever >38°C was observed, followed by wet cough on day 3, resulting in a positive PCR test indicating COVID‐19 infection. Chest computed tomography (CT) revealed multiple ground‐glass‐like patchy shadows throughout the entire bilateral lungs (Figure 1A,B). He received nasal cannula support at 2 L/min, intravenous dexamethasone (6 mg once daily) and antibiotic prophylaxis. His respiratory condition sharply deteriorated on postoperative day 7, requiring ventilation management. He underwent dialysis with a central venous catheter the same day. The tidal volume was large, so deep sedation was performed to suppress consequent lung injury, with positive end‐expiratory pressure set to approximately 8–10 cmH_2_O. However, the arterial oxygen partial pressure to fractional inspired oxygen (PaO_2_/FiO_2_; PF) ratio gradually deteriorated to 100 units, and granular shadows were observed throughout the entire lung field on chest x‐ray (Figure 2A). Because he developed severe hypoxaemic respiratory failure from acute respiratory distress syndrome (ARDS), 500 mg of methylprednisolone was infused intravenously daily for 3 days from postoperative day 10. Subsequently, his respiratory condition improved dramatically, and on postoperative day 14, the PF ratio was 400 units, while chest x‐ray showed marked improvement in permeability (Figure 2B). The patient was able to be weaned off the ventilator the same day. In the laboratory test, the C‐reactive protein value was high at 15.1 prior to the pulse therapy, but it dramatically decreased to 2.8 after 4 days of therapy. After the pulse therapy, the patient received treatment with oral corticosteroids (prednisone 40 mg once daily, 0.5 mg/kg, weight 80 kg). On postoperative day 20, oxygen administration became unnecessary, and quarantine was lifted on postoperative day 28, with the forearm graft cannulated for dialysis thereafter. Chest CT indicated only fibrosis in the upper left lobe (Figure 1C,D). After that, the dose of corticosteroids was decreased by 10 mg/day, every 2 weeks.

## DISCUSSION

The case fatality rate of COVID‐19 infection in patients suffering from ESRD is reportedly as high as 20%–30%.^1^, ^2^ The therapeutic agents currently approved in Japan are remdesivir, steroids and baricitinib; however, only steroid treatment is possible in patients suffering from ESRD. Although low‐dose corticosteroid therapy was performed at the moderate stage in this case, the respiratory condition deteriorated, eventually requiring ventilation management. Although haemodialysis was initiated, following the start of ventilator use, the patient developed severe hypoxaemic respiratory failure from ARDS, causing his condition to become extremely serious. Edalatifard et al.^3^ conducted a prospective study of pulse therapy in patients with severe COVID‐19 infection and showed that the mortality was significantly reduced in the steroid pulse group (5.9% in the administration group vs. 42.9% in the standard treatment group). Although this study was not conducted in patients suffering from ESRD, we suspected that steroid pulse might be effective in patients suffering from ESRD.

The case fatality rate of COVID‐19 patients during the perioperative period was 20%, indicating a very poor prognosis.^4^ In addition to ensuring patient safety, surgeons need to perform elective surgery and perioperative management while also considering minimizing the risk of infection among healthcare professionals. Surgical triage recommended by the American College of Surgeons is known to determine the implementation or postponement of elective surgery. Because this patient was suffering from severe ESRD, in addition to also suffering from respiratory failure, he was considered to be in stage 3, a state that required surgery be carefully conducted while taking sufficient infection prevention measures, so the operation itself was believed to be appropriate. PCR tests were performed at the time of admission to our hospital for all patients undergoing elective surgery in order to prevent infection, and the test for this patient was confirmed to be negative at that time. The present patient required oedema treatment and blood transfusion prior to surgery, which took 1 week to complete. It is highly possible that he became exposed to the virus while hospitalized after admission and developed the infection the day after surgery. The COVID‐19 infection was confirmed in his hospital roommates in the preoperative admission room, as well as in the staff who were active therein. Fortunately, the surgeon and the anaesthesiologist who conducted the therapy did not become infected with COVID‐19. The incubation period for COVID‐19 infections is said to be from 1 to 14 days. Furthermore, the incidence of asymptomatic infection is reportedly more common than with the flu, ranging from single‐digit percentages to 60%.^5^ Given these points, PCR testing should have been performed again in this patient immediately prior to the operation.

## CONFLICT OF INTEREST

None declared.

## AUTHOR CONTRIBUTION

Sokichi Kamata and Kiyohiro Fujiwara designed this report and wrote the manuscript. Sokichi Kamata and Souhei Hamanaka performed AVG surgery. Jin Tsukamoto, Teppei Tsuchimoto and Mikio Okamura contributed to the patient's therapy. All authors were involved in drafting the manuscript and approved the final version of the manuscript.

## ETHICS STATEMENT

The authors declare that appropriate written informed consent was obtained for the publication of this case report and accompanying images.
